# The Determination of Parkinson's Drugs in Human Urine by Applying Chemometric Methods

**DOI:** 10.1155/2019/7834362

**Published:** 2019-07-09

**Authors:** Guzide Pekcan Ertokus

**Affiliations:** Department of Chemistry, Faculty of Science & Art, Süleyman Demirel University, 32260 Isparta, Turkey

## Abstract

The spectrophotometric-chemometric analysis of levodopa and carbidopa that are used for Parkinson's disease was analyzed without any prior reservation. Parkinson's drugs in the urine sample of a healthy person (never used drugs in his life) were analyzed at the same time spectrophotometrically. The chemometric methods used were partial least squares regression (PLS) and principal component regression (PCR). PLS and PCR were successfully applied as chemometric determination of levodopa and carbidopa in human urine samples. A concentration set including binary mixtures of levodopa and carbidopa in 15 different combinations was randomly prepared in acetate buffer (pH 3.5).). UV spectrophotometry is a relatively inexpensive, reliable, and less time-consuming method. Minitab program was used for absorbance and concentration values. The normalization values for each active substance were good (r2>0.9997). Additionally, experimental data were validated statistically. The results of the analyses of the results revealed high recoveries and low standard deviations. Hence, the results encouraged us to apply the method to drug analysis. The proposed methods are highly sensitive and precise, and therefore they were implemented for the determination of the active substances in the urine sample of a healthy person in triumph.

## 1. Introduction

**Figure 1:**
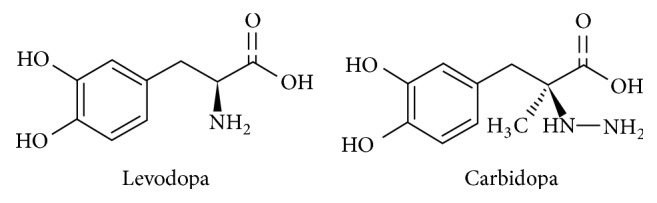
The chemical formulas of Parkinson's drugs.

Levodopa, which has the [(-)-3-(3,4-dihydroxyphenyl)-L-alanine] chemical expansion, is an antiparkinson drug and a dopamine precursor. It is often used in combination with carbidopa and acts as an amino acid decarboxylase inhibitor [1]. To treat Parkinson's disease, levodopa is returned to dopamine in the brain and readily crosses the blood barrier [2]. [(-)-L-2-(3,4-dihydroxy benzyl)-2-hydrazino propionic acid] is the chemical structure of carbidopa, another drug used in the treatment of this disease [3]. Carbidopa is a catechol related compound [4]. The chemical formulas of these compounds are indicated in Figure 1 [5].

When levodopa is combined with carbidopa, the side effects are normally reduced, and the concentration of dopamine is efficacious when these are given at an appropriate level [4].

Multivariate calibration methods (PLS-PCR) were developed using various mathematical operations to determine two or more compounds in the same sample without any chemical separation [6, 7]. The chemometric methods are utilized in the spectral data analysis in overlapping spectra containing two or more compounds [8]. Minitab 17 program, which is statistical analysis software (Anova, Ankara, Turkey), to determine concentration, allows quantitative analysis to evaluate absorbance data simultaneously [9]. Chemometric calibration methods are considered the best techniques to determine the amount of each component in complex mixtures. The most commonly accepted chemometric methods in drug analysis are principal component regression (PCR) and partial least squares regression (PLS) [10]. A relationship between matrices of chemical data can be determined using chemometric methods [11].

A concentration data matrix (y-block) and absorbance data matrix (x-block) were used to generate PLS and PCR calibration equations at the calibration stage. The first step in the chemometric calculations was a PCA, which allowed the calculation of the amount of available data given the original variables. Using the first few of the prediction variables instead of the components of the prediction variables reduced the number of new components (PC) created. The PLS and PCR were applied, providing precision, accuracy, and selectivity in the results.

Different analytical methods have been used in the literature to identify levodopa and carbidopa both separately and together. Moreover, there are several methods for diagnosing Parkinson's disease including spectrophotometric methods [1–14], electrochemical methods [15–22], chromatographic methods [23–52], ATR-FTIR spectrometric method [53], NMR [54], and capillary electrophoresis [55, 56].

In this study, we report analysis of levodopa and carbidopa in urine sample of a healthy person with chemometric methods. Levodopa and carbidopa are contained in the same drug used for treatment of Parkinson's disease. Our aim with this drug was to carry out quantitative analyses using chemometry in urine sample. Levodopa and carbidopa provide the same wavelength and overlapping spectra. Although it is difficult to analyze this situation by classical methods without any preseparation process, the chemometric methods and their determinations were successfully performed next to each other.

The validity of this method is clearly examined in terms of accuracy, precision, and selectivity. The chemometric methods used in the study were successfully carried out without any preseparation method for simultaneous determination of levodopa and carbidopa in urine sample of a healthy person. The results were statistically compared to each other and the validity of the methods according to the calculated analytical parameters (SD, RDS, PRESS, etc.) was evaluated.

## 2. Experimental

### 2.1. Materials

Stock solutions of 25 mg/250 mL levodopa (Sigma) and carbidopa (Sigma) in the analytical purity used in this study were prepared in acetate buffer (pH 3.5). Measurement of the synthetic mixture (for verification and calibration) in the concentration set prepared symmetrically from the active ingredients of the drugs was recorded with the UV-VİS-1700 PharmaSpec Spectrophotometer (Shimadzu, Kyoto, Japan).

### 2.2. Experimental Design and Sample Preparation

**Figure 2:**
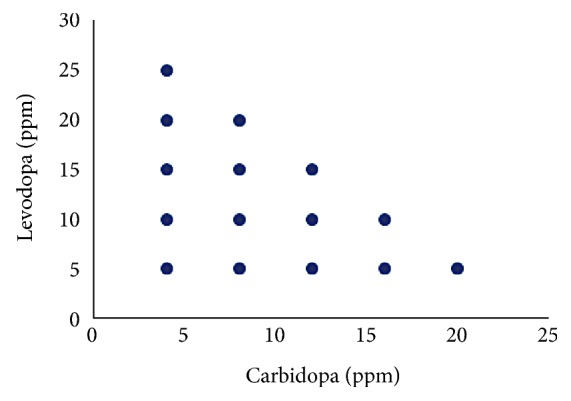
Design for concentration set.

Absorption spectra of levodopa and carbidopa were recorded at 0.1 ranges from 210 nm to 310 nm. The calibration matrix, training, and validation kits (Table 1 and Figure 2) contain a two-component mixture of different proportions. In the analysis of prepared synthetic mixtures and human urine samples, the data were calculated using PLS and PCR. 4.0 mg L^−1^ and 25.0 mg L^−1^ of levodopa and carbidopa, respectively, were placed in flasks (25 mL) and dissolved by adding acetate buffer to develop the experimental design. The partial factorial design was used in preparing the calibration set. Chemometric methods are based on suitable experimental design.

### 2.3. Procedure to Analyze Levodopa and Carbidopa in Urine Sample of a Healthy Person

Twenty-four-hour drug-free human urine samples were collected from healthy individuals and stored at -20°C before analysis. The urine samples were collected over a 4-h period in plastic containers before analysis. A healthy human urine sample was diluted with 20-fold deionized water to remove the matrix. Potentially interfering compounds need to be removed before analysis. Protein precipitation of urine samples was carried out by the addition of acetonitrile. After thawing to ambient temperature, 1 mL of urine was mixed with 9 mL of acetonitrile in order to precipitate proteins from the specimen, vortexed for 3 min. The resulting different (4-20 mg L^−1^) concentrations of levodopa and carbidopa were added to the analysis. In biological samples, matrix effects were taken into consideration and uv-visible measurements were taken. In the calculations of biological samples, the dilution process in the experimental stage was taken into consideration.

## 3. Results and Discussions

### 3.1. Absorption Spectra of Levodopa-Carbidopa Mixtures

**Figure 3:**
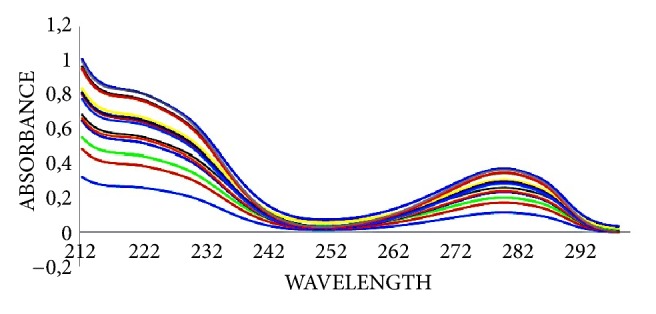
Absorption spectra of levodopa-carbidopa mixtures.

Absorption spectra of levodopa-carbidopa mixtures were in the range of 210 nm-310 nm (Figure 3).

When the absorbance-concentration plots for levodopa and carbidopa are examined, it is seen that the absorbance value increases as the concentration increases. The linear relationship [58] between absorbance and concentration was confirmed by the fact that the regression coefficient [59] is close to the individual values (Table 3).

### 3.2. Chemometric Methods (PLS and PCR)

Recovery (%), mean, and recovery value (%) (Table 2) were calculated from added and found values for levodopa-carbidopa mixtures.

### 3.3. Validation of the Method

The chemometric methods were validated on the basis of ICH rules [60–62] for linearity, accuracy, intraday and interday sensitivity, detection limit, and quantitative limit.

In order to verify the validity of the system, one of the calculated parameters is the PRESS (equation (1)) [63] (Table 3) value known as the predictive residual error. The difference between the actual and the estimated value is calculated from the sum of the squares. If the PRESS value is close to zero, it indicates that the method used is good. It is particularly useful in comparing the prediction power of different methods.

(1) $$\begin{array}{c} PRESS=\overset{n}{\underset{i=1}{\sum }}{\left(\;C_{i}^{added}-C_{i}^{found}\;\right)}^{2} \end{array}$$

*C*_*i*_^*added*^ is actual concentration, the added concentration of drug; *C*_*i*_^*found*^ is predicted concentration, the calculated concentration of drug.

Another validation parameter is RMSEC (equation (2)) [64].

(2) $$\begin{array}{c} RMSEC=\sqrt{\frac{PRESS}{n}} \end{array}$$

The observable limit (LOD) and the detection limit (LOQ) parameters are interrelated but have different definitions (equations (3) and (4)) [65].

(3) LOD=3Sam

(4) LOQ=10Sam

m is slope; LOQ > LOD and LOQ = LOD were taken into consideration while evaluating the calculated LOD values [66].

The limit of detection (LOD) is defined as the lowest concentration of an analyte in a sample that can be detected. The limit of quantification (LOQ) is defined as the lowest concentration of an analyte in a sample that can be determined with acceptable precision and accuracy under the stated operational conditions of the method (Table 3).

**Figure 4:**
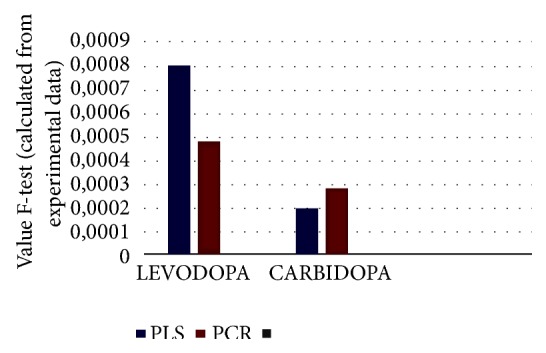
The results of the one-way ANOVA test according to Snedecor's* F*-test.

The values obtained from the two chemometric methods (PLS and PCR) used in this study were compared with the variance analysis-Snedecor's* F*-test [67]. Analysis of variance (ANOVA) is a powerful statistical technique used to test hypotheses about whether the difference between averages of two or more groups is significant. For PLS method, the F-test value for levodopa was 0.00080 and the p-value was 0.98 for the intergroup degrees of freedom = 1, and intragroup degrees of freedom = 28%. For the carbidopa, the F-test value was calculated as 0.00020 and the p-value was 0.99 for the intergroup degrees of freedom = 1, and intragroup degrees of freedom = 28%. In the PCR method, the F-test value for levodopa was calculated to be 0.00048 and p-value was 0.98 for the intergroup degrees of freedom = 1, and intragroup degrees of freedom = 28%. For the carbidopa, the F-test value was calculated as 0.00029 and the p-value was 0.99 for the intergroup degrees of freedom = 1, and intragroup degrees of freedom = 28% and 95% confidence interval (Figure 4).

### 3.4. Analysis of Human Urine Samples

The experimental results of the two methods for human urine samples are given in Table 4. One can see that the obtained results are very close to each other. The suggested methods were successfully applied for the determination of the drugs used in healthy human urine samples and yielded convincing results as shown in Table 4.

All statistical parameters and numeric values appear suitable for simultaneous identification of these drugs in human urine samples.

## 4. Conclusion

With the UV-VIS spectrophotometric method, the drug active ingredients in the healthy human urine sample were determined as fast and reliable. The results obtained from urine samples were compared with the results obtained by conventional UV-VIS spectrophotometry. In this study, the results were obtained with support from chemometric methods and the results were still more reliable. At the same time, due to the fact that each drug substance was determined simultaneously with chemometric methods, time and cost losses were prevented.

After certain processes, the proteins were precipitated from the urine, and measurements were taken in the UV-Visible region. The data obtained were evaluated chemometrically. The main advantage of the method is the ability to analyze simultaneously the two compounds under similar conditions. The achieved method validation results show the reliability of the method. The high recovery values also showed that the drugs did not bind to urine proteins. The results obtained suggest that this method could be used suitably for simultaneous determination of levodopa and carbidopa in urine.

## Figures and Tables

**Table 1:** Concentration set for levodopa and carbidopa.

No.	Concentration, mg L^−1^	
	Levodopa	Carbidopa
1	4	5
2	4	10
3	4	15
4	4	20
5	4	25
6	8	5
7	8	10
8	8	15
9	8	20
10	12	5
11	12	10
12	12	15
13	16	5
14	16	10
15	20	5

**Table 2:** Calculated statistical parameters by chemometric methods [57].

	Levodopa		Carbidopa	
Mix	Recovery %	Recovery %	Recovery %	Recovery %
No	PLS	PCR	PLS	PCR
1	99.86	99.75	99.8	100
2	99.50	99.25	99.7	99.6
3	97.25	99.50	99.73	99.27
4	99.25	100.00	99.85	99.7
5	98.75	97.25	99.8	99.84
6	98.00	99.75	99	99.8
7	99.38	99.63	99.6	99.5
8	99.38	99.38	99.67	99.8
9	98.63	99.50	99.75	99.9
10	99.50	99.58	99.8	98.4
11	99.67	99.00	98.9	99.4
12	99.33	99.67	99.93	99.73
13	99.75	99.88	99.2	99.8
14	99.69	99.75	99.8	99.8
15	99.88	99.80	99.6	99.4
	*Mean*=99.18	*Mean*=99.18	*Mean*=99.61	*Mean*=99.61
	*RSD*%=0.74	*RSD*%=0.74	*RSD*% =0.32	*RSD*% =0.32

**Table 3:** Validation parameters for chemometric determination of levodopa and carbidopa using PLS and PCR methods.

Parameters	Method	Levodopa	Carbidopa
*ƛ*_max_ (nm)		280 nm	280 nm

Correlation Coefficient (R^2^)		0.9997	0.9992

PRESS	PLS	0.004	0.002
	PCR	0.0001	0.002

RMSEC	PLS	4.21x10^−3^	9.42x10^−3^
	PCR	6.6x10^−4^	9.42x10^−3^

LOD (*μ*g/mL)	PLS	0.052043	0.04631
	PCR	0.04631	0.140332

LOQ (*μ*g/mL)	PLS	0.157706	0.052817
	PCR	0.140332	0.160051

Accuracy (% Recovery ± SD)	PLS	99.18 ± 0.74	99.61 ± 0.32
	PCR	99.18 ± 0.74	99.61 ± 0.32

Precision (Reproducibility)			

Intraday (% Recovery ± SD) (n:6)	PLS	99.65 ± 0.95	99.97 ± 0.64
	PCR	99.96 ± 0.75	99.98 ± 0.42

Interday (% Recovery ± SD) (n:6)	PLS	99.75 ± 0.30	98.95 ± 0.82
	PCR	98.98 ± 0.41	99.89 ± 0.81

**Table 4:** Determination of levodopa and carbidopa in human urine using PLS and PCR methods.

	Levodopa (PLS)			Carbidopa (PLS)		
Mix	Added	Found	Recovery	Added	Found	Recovery
No	(mg L^−1^)	(mg L^−1^)	(% mean)	(mg L^−1^)	(mg L^−1^)	(% mean)
1	4	3.98	99.50	4	3.97	99.25
2	8	7.98	99.75	8	7.96	99.50
3	12	11.96	99.67	12	11.97	99.75
4	16	15.98	99.88	16	15.99	99.94
5	20	19.89	99.45	20	19.98	99.90
Mean ± SD*∗*			99.65			99.67
Standard Deviation			0.177			0.29

	Levodopa (PCR)			Carbidopa (PCR)		
Mix	Added	Found	Recovery	Added	Found	Recovery
No	(mg L^−1^)	(mg L^−1^)	(% mean)	(mg L^−1^)	(mg L^−1^)	(% mean)

1	4	3.96	99.00	4	3.98	99.5
2	8	7.96	99.50	8	7.98	99.75
3	12	11.97	99.75	12	11.99	99.92
4	16	15.96	99.75	16	15.98	99.88
5	20	19.98	99.90	20	19.97	99.85
Mean			99.58			99.78
Standard Deviation			0.35			0.17

	Levodopa (Classical UV-Vis)			Carbidopa (Classical UV-Vis)		
Mix	Added	Found	Recovery	Added	Found	Recovery
No	(mg L^−1^)	(mg L^−1^)	(% mean)	(mg L^−1^)	(mg L^−1^)	(% mean)

1	4	3.97	99.25	4	3.95	98.75
2	8	7.95	99.38	8	7.99	99.88
3	12	11.95	99.58	12	11.96	99.67
4	16	15.93	99.56	16	15.98	99.88
5	20	19.99	99.95	20	19.96	99.80
Mean			99.54			99.60
Standard Deviation			0.26			0.48

## Data Availability

The data used to support the findings of this study are included within the article.
